# Hydration Mechanisms of Alkali-Activated Cementitious Materials with Ternary Solid Waste Composition

**DOI:** 10.3390/ma15103616

**Published:** 2022-05-18

**Authors:** Zhijie Yang, De Zhang, Chengyang Fang, Yang Jiao, Dong Kang, Changwang Yan, Ju Zhang

**Affiliations:** 1 School of Mining and Technology, Inner Mongolia University of Technology, Hohhot 010051, China; 20201100458@imut.edu.cn (D.Z.); 20211100472@imut.edu.cn (C.F.); 20211800659@imut.edu.cn (Y.J.); 20201100459@imut.edu.cn (D.K.); yanchangwang@imut.edu.cn (C.Y.); zj970741@imut.edu.cn (J.Z.); 2 The Key Laboratory of Green Development for Mineral Resources, Inner Mongolia University of Technology, Hohhot 010051, China

**Keywords:** CSS, FA, BFS, sodium silicate, alkali-activated, hydration mechanism

## Abstract

Considering the recent eco-friendly and efficient utilization of three kinds of solid waste, including calcium silicate slag (CSS), fly ash (FA), and blast-furnace slag (BFS), alkali-activated cementitious composite materials using these three waste products were prepared with varying content of sodium silicate solution. The hydration mechanisms of the cementitious materials were analyzed by X-ray diffraction, Fourier-transform infrared spectroscopy, scanning electron microscopy, and energy dispersive spectroscopy. The results show that the composite is a binary cementitious system composed of C(N)-A-S-H and C-S-H. Si and Al minerals in FA and BFS are depolymerized to form the Q^0^ structure of SiO_4_ and AlO_4_. Meanwhile, β-dicalcium silicate in CSS hydrates to form C-S-H and Ca(OH)_2_. Part of Ca(OH)_2_ reacts with the Q^0^ structure of AlO_4_ and SiO_4_ to produce lawsonite and wairakite with a low polymerization degree of the Si-O and Al-O bonds. With the participation of Na^+^, part of Ca(OH)_2_ reacts with the Q^0^ structure of AlO_4_ and the Q^3^ structure of SiO_4_, which comes from the sodium silicate solution. When the sodium silicate content is 9.2%, the macro properties of the composites effectively reach saturation. The compressive strength for composites with 9.2% sodium silicate was 23.7 and 35.9 MPa after curing for 7 and 28 days, respectively.

## 1. Introduction

Alkali-activated cementitious material is a kind of aluminosilicate polymer composed of AlO_4_ and SiO_4_ tetrahedrons, which are formed by intramolecular ionic and covalent bonds that are connected by intermolecular van der Waals forces [1]. Due to the many advantages, such as salt-alkali corrosion resistance, freeze resistance, carbonization resistance, high durability, wide source of raw materials, and low production cost, alkali-activated cementitious materials have become a popular research topic in the field of hydraulic cementitious materials over the past 20 years [2,3,4]. Calcium silicate slag (CSS) is a new type of secondary solid waste produced in the process of alumina extraction from fly ash (FA) [5], and 2 tons of CSS is discharged per ton of alumina production while consuming 2.5 tons of FA [6]. CSS contains a certain amount of sodium salt, which makes it challenging to use as the raw material for traditional cement [7]. However, FA and blast-furnace slag (BFS), which are both solid waste materials mainly composed of Si and Al elements, can be used together with CSS to prepare alkali-activated cementitious materials. This makes full use of the Si and Al properties of FA and BFS, and the sodium salt in CSS can be used as the activator. However, the content of sodium salt in CSS is small, which cannot satisfy the needs of composite preparation for alkali-activated cementitious materials. Therefore, a certain amount of other alkali-activators still need to be added. Among many alkali-activators, sodium silicate has superior activation effects. Many studies have investigated sodium silicate as an alkali-activator in cementitious materials.

Zhang Jinxi studied cementitious material activated by sodium silicate for application in red mud road base [8], and the main hydrates were C-S-H. Du Tianling studied the effect of sodium silicate on FA and BFS geopolymer, and the results showed that Na_2_O in sodium silicate dissolved Si and Al elements contained in FA and BFS, which then polymerized with SiO_2_ in sodium silicate again [9]. Huang Liping studied the alkali-activated cementitious materials prepared with BFS and sodium silicate, and the hydrates were C-S-H and C-A-S-H at low C/S molar ratio [10]. Shi Di researched the optimal ratio of alkali-activated cementitious materials prepared by CSS and BFS, and the results showed that compressive strength of alkali-activated cementitious materials can reach 25.0 MPa at 70% CSS and 30% BFS after curing for 28 days [11]. The hydration mechanisms and hydrates of alkali-activated cementitious materials are different when using different raw materials, which is different from ordinary Portland cement. Furthermore, there are drastic differences between hydrates of alkali-activated cementitious materials with varying curing time, such as Si/Al and Ca/Si for example [12,13,14].

Although previous studies have considered the preparation of alkali-activated cementitious materials using sodium silicate to activate a single solid waste, there is little research on the preparation using sodium silicate to activate three solid wastes such as CSS, FA, and BFS together. Therefore, to effectively utilize CSS, FA, and BFS, experiments of alkali-activated cementitious materials prepared with a ternary solid waste composition were conducted using varying content of sodium silicate. The composites were analyzed by X-ray diffraction (XRD), Fourier-transform infrared (FTIR) spectroscopy, scanning electron microscopy (SEM), and energy dispersive spectroscopy (EDS) after curing for 7 and 28 days. For this report, the hydration reaction mechanisms are discussed in detail.

## 2. Materials and Methods

### 2.1. Materials

The main raw materials used in this experiment are CSS, FA, and BFS. The CSS is from the China Datang International Renewable Resources Co., Ltd., Hohhot, Inner Mongolia, China, which has the world’s first production line of alumina extraction from FA. The BFS is from Baotou Iron and Steel Co., Ltd., Baotou, Inner Mongolia, China. The FA is from China Datang International Tuoketuo plant Co., Ltd., Hohhot, Inner Mongolia, China. The chemical constituents of the raw materials were analyzed by X-ray fluorescence (Shimadzu, Japan, XRF-1800), and the results are shown in Table 1 below.

**Figure 1:**
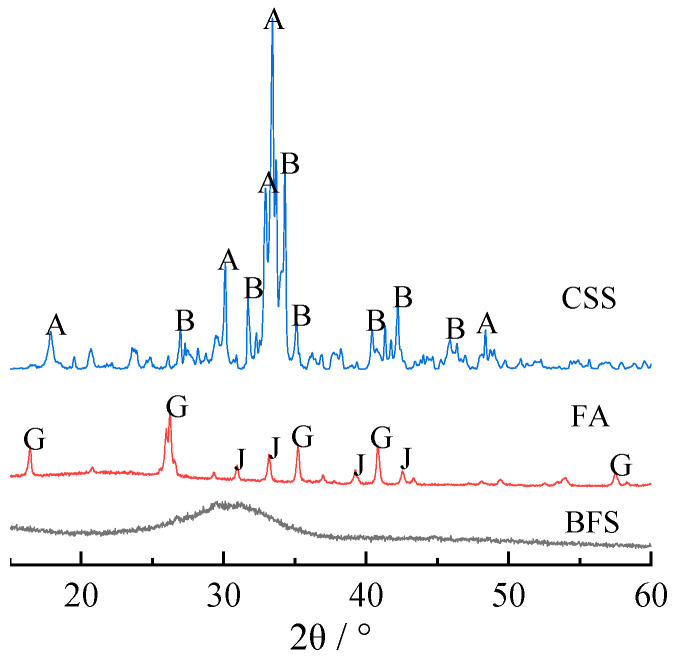
XRD patterns of raw materials. A, β-dicalcium silicate (β-2CaO·SiO_2_); B, calcite (CaCO_3_); G, mullite (3Al_2_O_3_·2SiO_2_); J, quartz (SiO_2_).

The phase composition of raw materials was analyzed by X-ray diffraction. As shown in Figure 1, the main phases of CSS are β-dicalcium silicate and calcite. The main phase of BFS is the glass phase. The main phases of FA are mullite and quartz. Other raw materials used in the experiment include alkali-activator, distilled water, and absolute ethanol. The alkali-activator is sodium silicate solution with a modulus of 2.4 and density of 1371.8 g/L.

### 2.2. Methods

First, the CSS, BFS, and FA were dried to constant weight in an oven at 110 °C, and then each raw material was put into a 5 kg cement test mill for grinding and to control the specific surface area at about 450 m^2^/kg. Then, each material was weighed and mixed in a planetary mixer for 5 min according to the mass fractions of 40% CSS, 40% FA, and 20% BFS. In each experiment, 450 g of the mixture was weighed, and sodium silicate solution and distilled water were mixed according to Table 2 to ensure the water-cement ratio of 0.5. Composite specimens were prepared and formed in a 40 mm × 40 mm × 40 mm mold. Then, test specimens were cured in a cement standard curing box (temperature 20 ± 1 °C, humidity ≥90%), followed by demolding 24 h after curing. After curing for 7 or 28 days, the compressive strength of the specimens was tested using a cement constant stress instrument (DYE-300s), and the specimen debris was submerged in anhydrous ethanol for 24 h to terminate hydration.

Composite specimens were dried to constant weight in the oven at 50 °C and ground to 300 mesh size. Finally, the phase composition, chemical structure, and micromorphology of the materials were analyzed using an X-ray diffractometer (PANalytical, Netherlands, X’Pert Powder 3, Cu target, 40 kV, scanning range 10−100°, step 0.02°), scanning electron microscope (Hitachi S-4800), and FTIR spectrometer (VERTEX 70, Bruke, Germany, resolution: 4 cm^−1^, scanning times: 32, wavenumber range: 4000–400 cm^−1^).

The setting time of the composites was tested according to the standard GB/T 1346-2011 (Test methods for water requirement of normal consistency, setting time, and soundness of the Portland cement).

## 3. Results and Discussion

### 3.1. Phase Analysis

**Figure 2:**
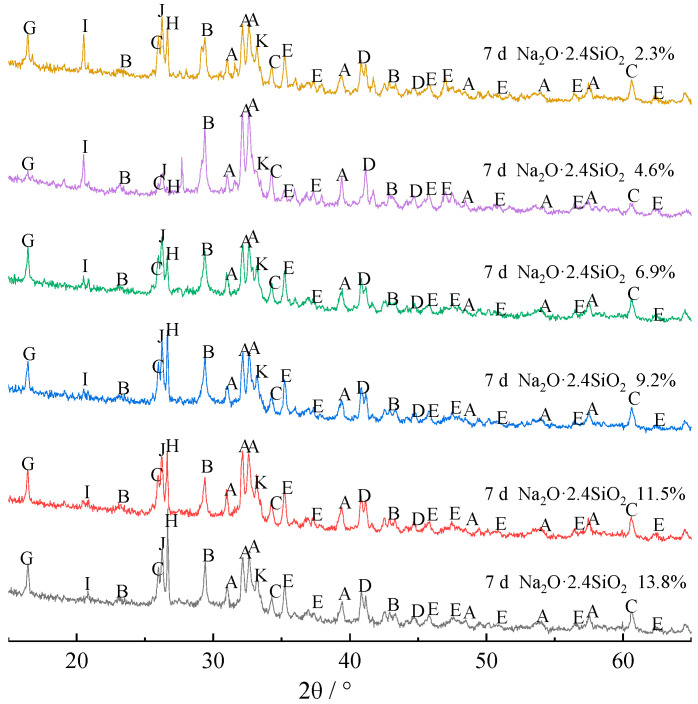
XRD patterns of composite alkali-activated cementitious materials after curing for 7 days. A, β-dicalcium silicate (β-2CaO·SiO_2_); B, calcite (CaCO_3_); C, lawsonite (CaO·Al_2_O_3_·2SiO_2_·2H_2_O); D, C-S-H (xCaO·SiO_2_·(m+x−2)H_2_O); E, boggsite (Na_3.7_Ca_7.4_Al_18.5_Si_77.5_O_192_·74H_2_O); G, mullite (3Al_2_O_3_·2SiO_2_); H, tetranatrolite ((Na,Ca)_2_(Si,Al)_5_O_10_·2H_2_O); I, beidellite (Na_0.3_Al_2_(Si,Al)_4_O_10_(OH)_2_·2H_2_O); J, quartz (SiO_2_); K, wairakite (CaO·Al_2_O_3_·4SiO_2_·2H_2_O).

**Figure 3:**
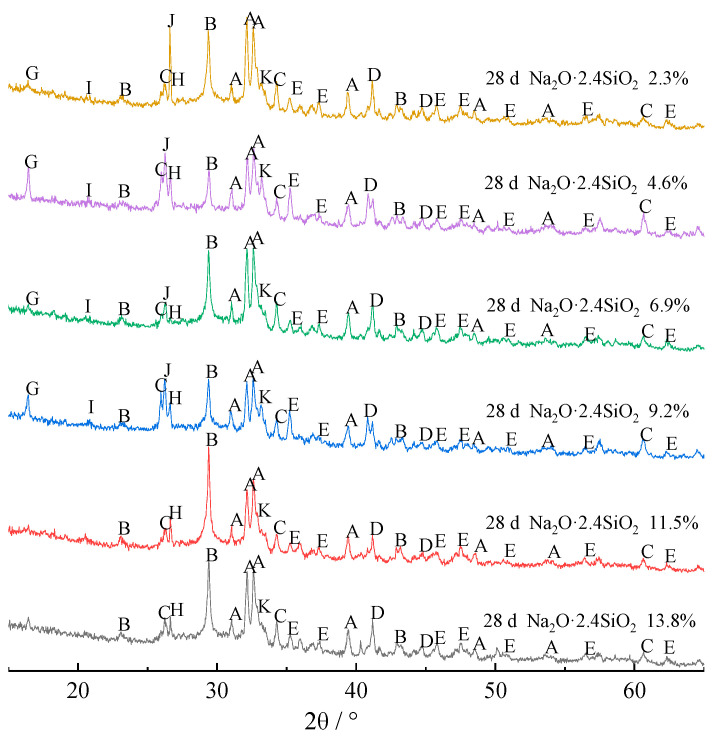
XRD patterns of composite alkali-activated cementitious materials after curing for 28 days. A, β-dicalcium silicate (β-2CaO·SiO_2_); B, calcite (CaCO_3_); C, lawsonite (CaO·Al_2_O_3_·2SiO_2_·2H_2_O); D, C-S-H (xCaO·SiO_2_·(m+x−2)H_2_O); E, boggsite (Na_3.7_Ca_7.4_Al_18.5_Si_77.5_O_192_·74H_2_O); G, mullite (3Al_2_O_3_·2SiO_2_); H, tetranatrolite ((Na,Ca)_2_(Si,Al)_5_O_10_·2H_2_O); I, beidellite (Na_0.3_Al_2_(Si,Al)_4_O_10_(OH)_2_·2H_2_O); J, quartz (SiO_2_); K, wairakite (CaO·Al_2_O_3_·4SiO_2_·2H_2_O).

To clarify the hydration mechanism and hydrate phase change rule of composite alkali-activated cementitious materials, the hydrates at 7 and 28 days were analyzed by XRD, and the results are shown in Figure 2 and Figure 3, respectively.

As seen in Figure 2, the main hydrates of the composite materials with different sodium silicate dosages at 7 days are β-dicalcium silicate, calcite, lawsonite, C-S-H, boggsite, mullite, tetranatrolite, beidellite, quartz, and wairakite. As a hydration mineral, the hydration reaction of β-dicalcium silicate is shown in Equation (1), and the main hydrates are C-S-H and Ca(OH)_2_. C-S-H in the hydrates comes from the hydration of β-dicalcium silicate. However, since β-dicalcium silicate has a relatively slow hydration rate, its 28 day hydration degree is only 10.3% [15]. Thus, there is still a large amount of unhydrated β-dicalcium silicate in the composite materials. The Ca(OH)_2_ generated by the hydration of β-dicalcium silicate and sodium silicate solution creates a strong alkaline environment. Under the polarization of OH^−^, the Si-O bond and Al-O bond in FA and BFS depolymerize to form AlO_4_ and SiO_4_ with Q^0^ structure, while SiO_4_ in 2.4 modulus sodium silicate solution is mainly Q^3^ structure [16]. Therefore, there are two SiO_4_ structures, Q^0^ and Q^3^, that may be found in the alkali-activated cementitious materials. This also allows for three main reactions of Ca(OH)_2_. Part of Ca(OH)_2_ reacts with the Q^0^ structure of AlO_4_ and SiO_4_ to produce lawsonite and wairakite with a low polymerization degree of the Si-O and Al-O bonds. With the participation of Na^+^ from sodium silicate solution, part of Ca(OH)_2_ reacts with the Q^0^ structure of AlO_4_ and Q^3^ structure of SiO_4_. Furthermore, a small part of Ca(OH)_2_ carbonizes with CO_2_ in the air to form calcite, which is a mineral that is difficult to undergo hydration. Therefore, the calcite in the composites contains the calcite generated by carbonization and the original calcite from CSS.

(1) $$2\mathrm{CaO}\cdot {\mathrm{SiO}}_{2}+{\mathrm{mH}}_{2}O=\mathrm{xCaO}\cdot {\mathrm{SiO}}_{2}\cdot \left(m+x-2\right)H_{2}O+\left(2-x\right)\mathrm{Ca}{\left(\mathrm{OH}\right)}_{2}$$

According to Figure 1 and Table 1, some of the Si and Al comes from FA in the mullite, quartz, and glass phases, and some comes from BFS in mainly the glass phase. The glass phase is metastable and more easily reacts with Ca(OH)_2_ and sodium silicate solution compared with crystal phases [17]. Therefore, the mullite and quartz phases in the composites are from the original mullite and quartz phases in FA.

At the same time, Figure 2 shows that the diffraction peak of the tetranatrolite phase gradually increases with the increase of the sodium silicate content, while the diffraction peak of the beidellite phase gradually decreases. This result indicates that the hydration reaction of these composites does not generate the beidellite phase more easily with the increase of the sodium silicate content.

Figure 3 shows that when the content of sodium silicate is less than 9.2%, the hydrates at 7 and 28 days are basically the same. However, when the content of sodium silicate is higher than 9.2%, mullite and beidellite disappear from the hydrates by day 28. The main reason is that, although the Si-O and Al-O bonds of crystalline minerals are not easy to depolymerize, the Si-O and Al-O bonds of mullite will gradually depolymerize under the strong alkaline environment. Thus, the mullite phase dissolves and disappears. However, in the process of polycondensation and regeneration of the -Si-O-Si(Al)- structure, since Al^3+^ is three-coordinated, Na^+^ is needed for coordination supplement in the process of forming a four-coordinated network structure with Si^4+^ [18]. Moreover, since the radius of Na^+^ is larger than that of Ca^2+^, Na^+^ is often replaced by Ca^2+^ [19], and the beidellite phase with low Na^+^ content also disappears.

### 3.2. Chemical Structure Analysis

**Figure 4:**
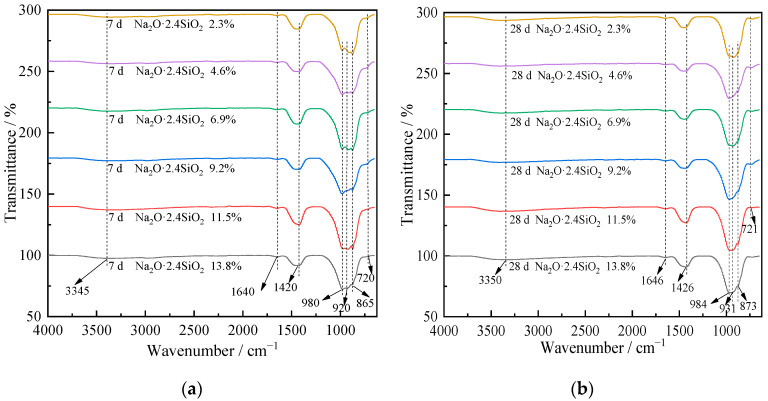
FTIR spectroscopy of composite alkali-activated cementitious materials. (**a**) FTIR spectroscopy after curing for 7 days (**b**) FTIR spectroscopy after curing for 28 days.

To further characterize the hydrate chemical structures of the composites after hydration for 7 and 28 days, FTIR spectroscopy was used to analyze the infrared spectrum. The results are shown in Figure 4. Figure 4a is analyzed according to Table 3, which shows various absorption bands corresponding to chemical group vibrations. The wavenumbers at 3345 and 1640 cm^−1^ correspond to the vibration characteristics of water molecules, but the band peak is not obvious due to the low moisture content of the specimens after drying. The wavenumber at 1420 cm^−1^ correspond to the asymmetric stretching vibration of CO_3_^2^^−^, which is due to the original calcite mineral in CSS and the calcite mineral formed by the carbonation of Ca(OH)_2_ generated from the hydration of CSS with CO_2_ in the air. The wavenumber at 980 cm^−1^ corresponds to the asymmetric stretching vibration of C-S-H, which reflects the existence of the hydrated calcium silicate phase in hydrates at 7 days. The wavenumbers at 920 cm^−1^ corresponds to the asymmetric stretching vibration of -Si-O-Si(Al)-. The wavenumber at 865 cm^−1^ corresponds to the bending vibration of -Si (Al)-OH. The wavenumber at 720 cm^−1^ corresponds to the bending vibration of-Si-O-Si (Al)-. This indicates that the sodium silicate solution and the sodium salt contained in the CSS promote the formation of OH^−^ during hydration. These OH^−^ ions can break the -Si-O-Si (Al)- bonding in the Si- and Al-based minerals of the raw materials. Then, further polycondensation occurs to generate -Si-O-Si (Al)- and -Si(Al)-OH structures, finally forming new C(N)-A-S-H hydrates, which corresponds with the XRD analysis.

Comparing Figure 4a,b, the infrared spectrums of hydrates at 7 and 28 days are basically similar. This result indicates that the types of hydrates in the composites are basically similar for the different curing ages. The main hydrates are C-S-H and C(N)-A-S-H. However, only the general band slightly shifts in the high frequency direction with the extension of curing time, which indicates that the polymerization degree of SiO_4_ and AlO_4_ tetrahedrons increases gradually with increasing curing time.

### 3.3. Micromorphology Analysis

**Figure 5:**
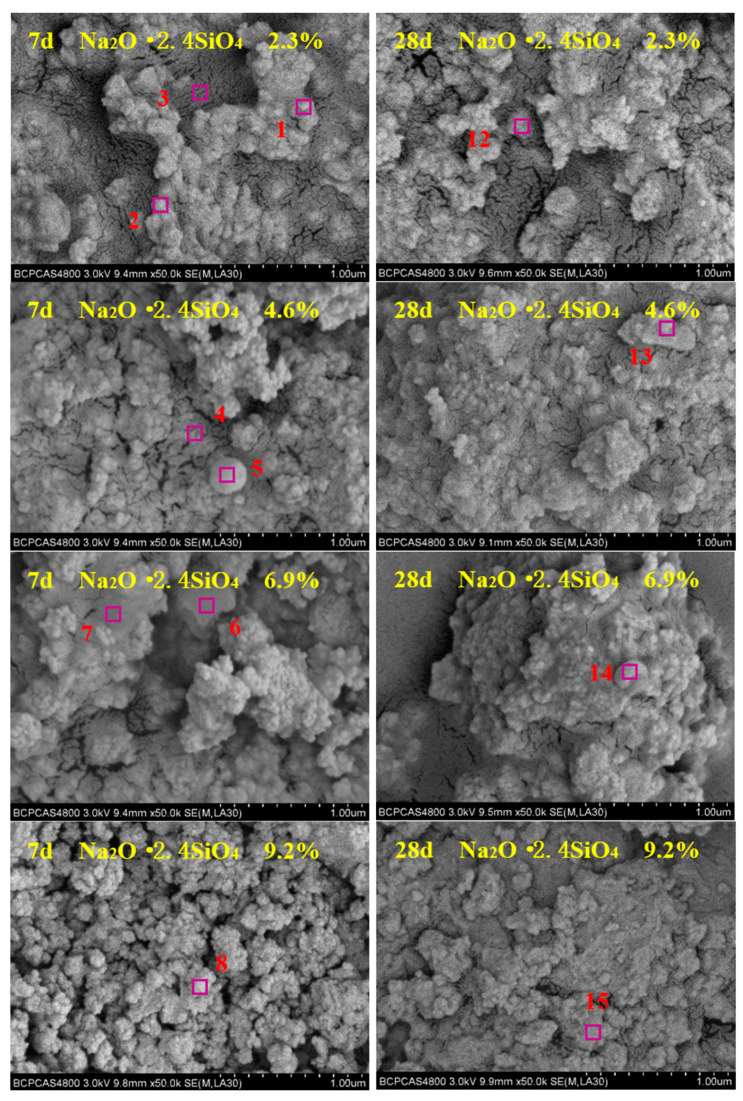

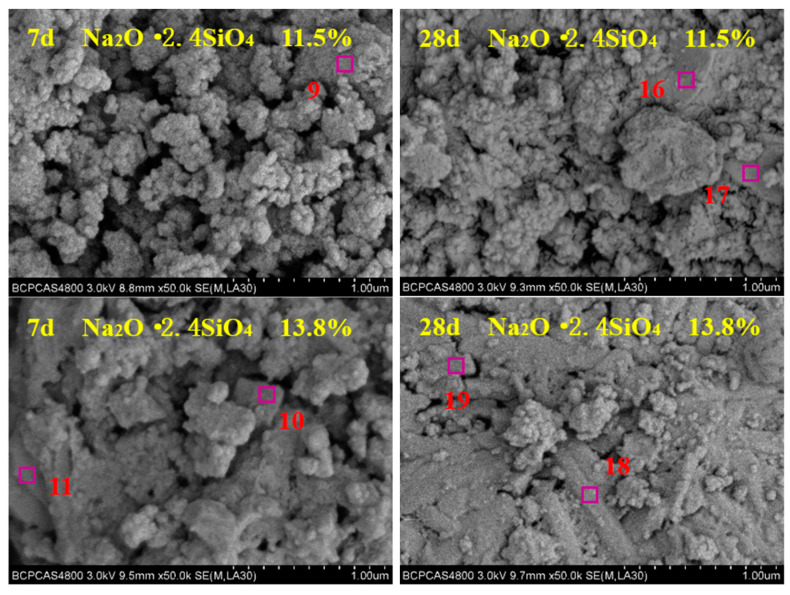
SEM images of composite alkali-activated cementitious materials after curing for 7 and 28 days.

The micromorphology of the composites was observed using a Hitachi S-4800 scanning electron microscope, and some special areas were tested by EDS. SEM images with 50,000× magnification are shown in Figure 5. According to the atomic ratio for different element, in combination with the XRD and FTIR analysis results, the corresponding phase of each region was identified, and the results are shown in Table 4.

From analysis of the SEM images, the granular-like β-dicalcium silicate and spherical-like mullite phases decreased significantly after curing for 28 days. This result is due to the continuous self-hydration of β-dicalcium silicate with the extension of curing time, and the alkali-activated hydration reaction of mullite with sodium silicate solution and Ca(OH)_2_. Moreover, the micromorphology of composites at 28 days is more uniform and denser than at 7 days. The composites at 7 and 28 days both exhibit more uniform and dense micromorphology with the increase of sodium silicate content.

### 3.4. Macro Properties Analysis

**Figure 6:**
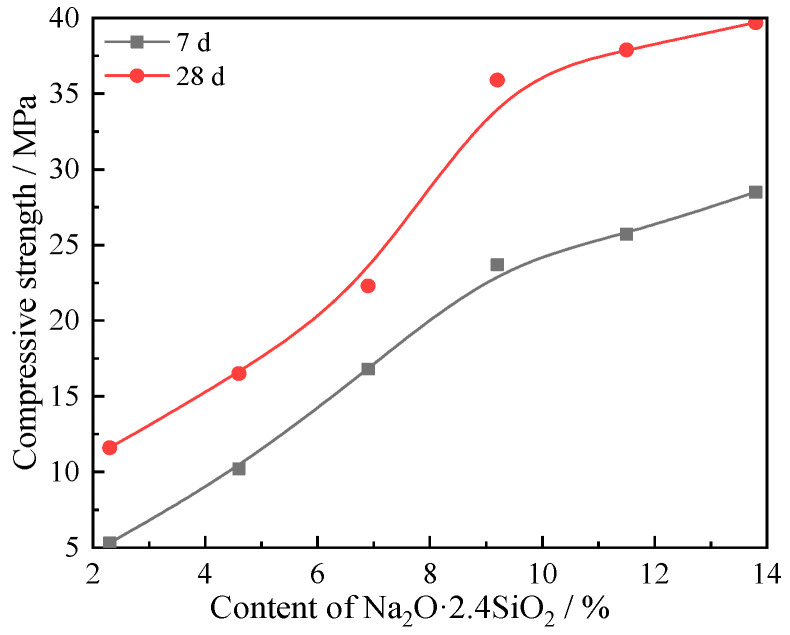
Cement paste compressive strength of composite alkali-activated cementitious material.

**Figure 7:**
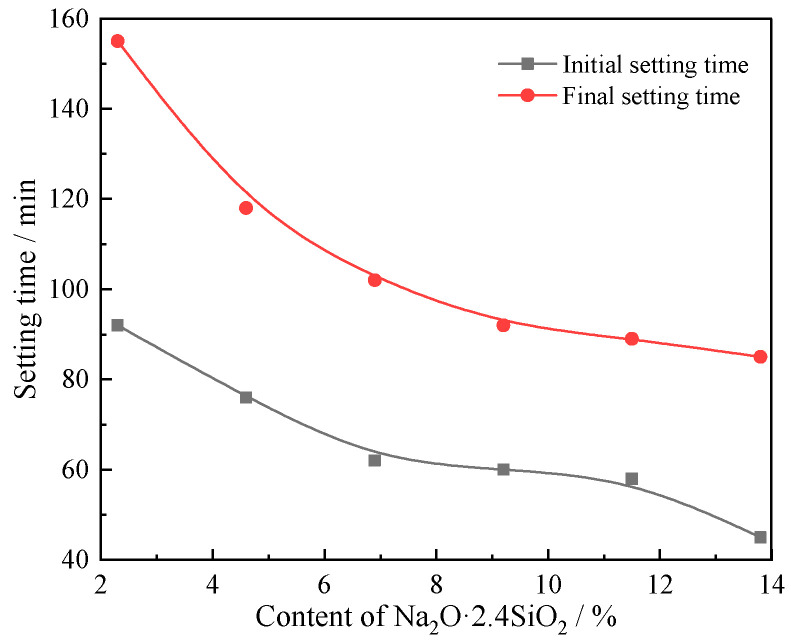
Setting time of composite alkali-activated cementitious materials.

The change of cement paste compressive strength and setting time of the composites as a function of sodium silicate content are shown in Figure 6 and Figure 7, respectively. With increasing sodium silicate content, the compressive strength increases, while the setting time decreases. There is a significant shift in the rates of change for the compressive strength and setting time depending on sodium silicate content. When the sodium silicate content is lower than 9.2%, the strength and setting time increase rapidly, or relatively sharply. When the sodium silicate content is higher than 9.2%, the rate of change for compressive strength and setting time slows down, becoming more gradual. When the content of compressive solution is 9.2%, the compressive strength at 7 and 28 days are 23.7 and 35.9 MPa, respectively, and the initial setting time and final setting time are 60 and 92 min, respectively.

Accordingly, when the content of sodium silicate is lower than 9.2%, unreacted mullite phase remains in the composite. With the increase of the sodium silicate content, the unreacted mullite will continue to undergo alkali-activated hydration. When the sodium silicate content is higher than 9.2%, the original Si- and Al-based minerals are almost completely reacted.

## 4. Conclusions

We prepared alkali-activated cementitious materials with a ternary solid waste composition using varying content of sodium silicate, and investigated the resulting compressive strength and setting time. The composites were analyzed by XRD, FTIR, SEM, and EDS after curing for 7 and 28 days. The hydration reaction mechanisms of the composites were discussed. The four major conclusions of this report are summarized as follows:

(1) From the hydration process, the alkali-activated cementitious composites contain Q^0^ and Q^3^ structural SiO_4_, in which Q^3^ structural SiO_4_ comes from the sodium silicate alkali-activator with a modulus of 2.4, while Q^0^ structural SiO_4_ comes from Si-O and Al-O bonds of FA and BFS, which are depolymerized under the polarization of OH^−^ in a strong alkaline environment to form Q^0^ structural AlO_4_ and SiO_4_.
(2) The alkali-activated cementitious material is a binary composite system composed of C(N)-A-S-H and C-S-H. The β-dicalcium silicate in CSS hydrates to form C-S-H and Ca(OH)_2_. There are three main reactions of Ca (OH)_2_. Part of Ca(OH)_2_ reacts with Q^0^ structural AlO_4_ and SiO_4_ to produce lawsonite and wairakite with a low polymerization degree of the Si-O and Al-O bonds, and part of Ca(OH)_2_ reacts with Q^0^ structural AlO_4_ and Q^3^ structural SiO_4_ with the participation of Na^+^ from the sodium silicate solution. A small part of Ca(OH)_2_ carbonizes with CO_2_ in the air to form calcite.
(3) Due to the metastable state of glass phases, the glass phase is more likely than the crystalline phase to be modified during the hydration reaction. A large amount of mullite remained in hydrates with limited alkali-activated hydration reaction. However, with prolonged curing time, and when the sodium silicate content is more than 9.2%, the mullite and beidellite disappear.
(4) With increasing sodium silicate content, the hydrate micromorphology at 7 and 28 days both become more uniform and dense, and the compressive strength increases, while the setting time decreases. When the content of sodium silicate is 9.2%, the macro properties of the composites effectively reach saturation. The compressive strength for composites with 9.2% sodium silicate was 23.7 and 35.9 MPa at 7 and 28 days, respectively, and the initial setting time and final setting time were 60 and 92 min, respectively.

## Figures and Tables

**Table 1:** The chemical constituents of raw materials (calculated by mass fraction %).

Constituents	SiO_2_	Fe_2_O_3_	Al_2_O_3_	CaO	MgO	Na_2_O	K_2_O	SO_3_	P_2_O_5_	F	Cl
CSS	31.08	2.25	5.97	50.35	3.61	2.31	0.36	3.21	0.42	0.15	0.29
BFS	34.57	0.51	10.50	42.75	4.13	0.77	0.46	2.78	3.34	0.12	0.07
FA	42.67	2.57	42.36	4.30	3.20	0.58	0.39	1.27	1.46	0.47	0.73

**Table 2:** Dosage of sodium silicate solution and distilled water for different composite alkali-activated cementitious materials.

Serial Number	Proportion of Na_2_O_2_·4SiO_2_ (wt%)	Na_2_O_2_·4SiO_2_ (mL)	Distilled Water (mL)
1	13.8	186	58
2	11.5	155	86
3	9.2	124	114
4	6.9	93	142
5	4.6	62	170
6	2.3	31	197

**Table 3:** Various absorption bands corresponding with chemical group vibrations [20,21].

Wavenumber/cm^−1^	Chemical Group Vibration
3450–3000	Stretching vibration of the water molecule
1650–1600	Bending vibration of the water molecule
1450–1400	Asymmetric stretching vibration of CO_3_^2^^−^
975–965	Asymmetric stretching vibration of C-S-H
950–900	Asymmetric stretching vibration of -Si-O-Si(Al)-
890–850	Bending vibration of -Si(Al)-OH
730–710	Bending vibration of -Si-O-Si(Al)-

**Table 4:** EDS analysis of composite alkali-activated cementitious materials and the corresponding phases.

Region	Micromorphology	Atomic Molar Ratio (at%)								Corresponding Phase
		O	Al	Si	Ca	Na	Mg	Fe	C	
1	granular-like	55.31	2.21	14.22	26.31	0.50	1.22	0.23	-	β-Dicalcium silicate
2	granular-like	54.56	1.75	15.42	26.22	0.52	1.13	0.40	-	β-Dicalcium silicate
3	worm-like	68.97	14.61	14.36	0.22	1.63	0.10	0.11	-	Beidellite
4	worm-like	69.38	14.82	14.29	0.11	1.19	0.12	0.09	-	Beidellite
5	spherical-like	60.46	27.71	10.52	-	0.27	-	1.04	-	Mullite
6	flake-like	63.15	0.42	17.78	16.52	-	0.81	1.32	-	C-S-H
7	flocculent-like	70.21	5.02	20.64	2.03	2.10	-	-	-	Boggsite
8	block-like	59.03	0.22	0.10	20.22	-	0.10	0.10	20.23	Calcite
9	flocculent-like	70.15	5.41	20.21	2.13	2.10	-	-	-	Boggsite
10	block-like	58.56	0.33	0.21	20.32	-	0.11	0.33	20.14	Calcite
11	strip-like	63.23	5.01	21.01	5.23	5.21	0.11	0.20	-	Tobermorite
12	flocculent-like	70.41	5.21	20.15	2.13	2.10	-	-	-	Boggsite
13	flake-like	65.95	0.23	16.72	16.33	0.12	0.44	0.21	-	C-S-H
14	spherical-like	62.48	25.85	9.96	-	0.59	0.34	0.78	-	Mullite
15	columnar-like	66.05	0.05	33.63	-	0.27	-	-	-	Quartz
16	layer-like	56.08	12.50	25.05	6.20	0.05	0.11	0.01	-	Wairakite
17	flake-like	66.12	0.23	16.55	16.33	0.12	0.44	0.21	-	C-S-H
18	strip-like	62.68	5.97	19.52	5.86	5.44	0.32	0.21	-	Tobermorite
19	strip-like	62.57	5.68	19.84	5.96	5.42	0.32	0.21	-	Tobermorite

## Data Availability

The data presented in this study are available on request from the corresponding author. At the time the project was carried out, there was no obligation to make the data publicly available.
